# Surface Wetting Behaviors of Hydroxyl-Terminated Polybutadiene: Molecular Mechanism and Modulation

**DOI:** 10.3390/polym16213085

**Published:** 2024-10-31

**Authors:** Xinke Zhang, Zhikun Liu, Bing Yuan, Kai Yang

**Affiliations:** 1 Center for Soft Condensed Matter Physics and Interdisciplinary, Research & School of Physical Science and Technology, Soochow University, Suzhou 215006, China; xkzhang@suda.edu.cn (X.Z.); l15945872422@163.com (Z.L.); 2 Songshan Lake Materials Laboratory, Dongguan 523808, China; 3 Jiangsu Key Laboratory of Frontier Material Physics and Devices, Suzhou 215006, China

**Keywords:** HTPB, surface wetting, entropy–enthalpy competition, polymer adsorption

## Abstract

The surface wetting or coating of materials by polymers is crucial for designing functional interfaces and various industrial applications. However, the underlying mechanisms remain elusive. In this study, the wetting behavior of hydroxyl-terminated polybutadiene (HTPB) on a quartz surface was systematically investigated using computer simulation methods. A notable tip-dominant surface adsorption mode of HTPB was identified, where the hydroxyl group at the end of the polymer chain binds to the surface to initiate the wetting process. Moreover, it was found that with the increase in the degree of polymerization (e.g., from DP = 10 to 30), spontaneous adsorption of HTPB becomes increasingly difficult, with a three-fold increase in the adsorption time. These results suggest a competition mechanism between enthalpy (e.g., adhesion between the polymer and the surface) and entropy (e.g., conformational changes in polymer chains) that underlies the wetting behavior of HTPB. Based on this mechanism, two strategies were employed: altering the degree of polymerization of HTPB and/or regulating the amount of interfacial water molecules (e.g., above or below the threshold amount of 350 on a 10 × 10 nm^2^ surface). These strategies effectively modulate HTPB’s surface wetting process. This study provides valuable insights into the mechanisms underlying the surface adsorption behavior of HTPB and offers guidance for manipulating polymer wetting processes at interfaces.

## 1. Introduction

The wetting of material surfaces by polymer molecules is a fundamental phenomenon with significant implications in various functional areas, including biological science (e.g., protein fibrillation at the condensate) and extensive industrial processes (e.g., polymeric coatings, separation, and adhesives) [1,2,3,4,5,6]. For instance, engineered superhydrophobic polymer-coated surfaces have been designed to exhibit exceptional properties such as water collection, drag reduction, heat transfer enhancement, and corrosion resistance, among others [6]. However, the molecular intricacies and underlying mechanisms governing the surface wetting of polymers remain elusive due to its occurrence at multiple spatial and temporal scales. Moreover, this process is further influenced by the physicochemical properties of polymers (e.g., chain structure, length, and monomer chemistry) as well as material surface characteristics. Therefore, substantial efforts are still required for a comprehensive understanding of the surface wetting behaviors of polymers.

Hydroxyl-terminated polybutadiene (HTPB) is a prominent telechelic liquid rubber with the ability to form a three-dimensional cross-linked network on the surfaces of various solid particles, thereby exhibiting distinctive characteristics such as high acid and alkali resistance, hydrolytic stability, exceptional abrasion resistance, and outstanding mechanical properties [7,8,9,10,11]. Therefore, HTPB finds particular application in the field of energetic materials with high sensitivity where its polymeric surface wetting and elastomeric network formation can absorb impact energy and mitigate mechanical insults, effectively reducing material sensitivity [12,13,14,15,16,17,18,19,20,21,22,23,24]. Nevertheless, the comprehension of the underlying mechanisms governing the surface wetting behavior of HTPB remains a significant challenge at both theoretical and practical levels.

In physics, the properties of polymer-based systems are predominantly determined by the interplay between enthalpy and entropy. For instance, it has been extensively demonstrated that enthalpy plays a pivotal role in determining the stabilization between layers within polymer-bonded explosives, the stability of self-assembled droplets in microemulsions, and the efficiency of membranes used for separating emulsified oil droplets [25,26]. On the other hand, the influence of entropy is more intricate [27,28]. For example, it has been found that conformational entropy transitions of molecules decorated in solid-state nano-channels have the potential to modulate ionic current, thereby providing an effective approach for high-resolution and highly controllable DNA sequencing [29]. The configurational entropy of membrane molecules maintains the structural integrity and controls the permeability of cell membranes, particularly at elevated temperatures [30,31]. Surface functionalization of materials is also a crucial approach for regulating the entropy and enthalpy properties of interfaces. For instance, efforts have been made to modify interfacial hydrogen bonding and van der Waals interactions through functionalized modifications in order to manipulate the hydrophilicity of pulverized coal surfaces [32]. Furthermore, precise detection of drug molecules has been achieved by adjusting the wettability of outer/inner functionalized nano-surfaces [33,34]. Overall, gaining insight into this complex competition between enthalpy and entropy is essential for understanding a polymer’s surface wetting behaviors. However, previous studies have mainly focused on optimizing interfacial wetting through interaction modulation (e.g., surface modification [35] and functionalization [24,32]) with a descriptive approach that explains spatial distribution and intermolecular disentanglement after wetting [36,37]. The study of how subtle conformational changes affect polymer wettability by hindering entropy variation remains underexplored. Therefore, it is urgent to investigate the HTPB molecule’s wetting properties from an entropy–enthalpy competition perspective with molecular resolution.

Computer simulation is an indispensable tool for investigating this issue, offering a more precise resolution of intricate inter- and intramolecular interactions at various interfaces and an in-depth analysis of the underlying mechanisms [38,39,40]. In this study, the interplay between conformational entropy and adsorption enthalpy in the wetting process of HTPB on a quartz surface was systematically examined through MD simulations. Specifically, a notable tip-dominant surface adsorption mode of HTPB was identified as the initiator of the wetting process. The degree of polymerization of HTPB and the number of interfacial water molecules were investigated as potential control variables for the entropy–enthalpy competition in the system. These findings contribute to a comprehensive understanding of the underlying mechanism governing the surface wetting of HTPB and offer potential strategies for manipulating this behavior for practical applications.

## 2. Materials and Methods

The MD simulations were performed using the Gromacs 2019.6 simulation package [41], while the VMD 1.9.3 software [42] was utilized for result visualization. The generalized Amber force field (GAFF) was employed to elucidate the bond and non-bond interactions between HTPB and the quartz surface [43]. Specifically, the van der Waals interactions were described by a 12-6 Lennard-Jones potential with a real space cut-off distance of 1.2 nm. An MMFF94 atomic charge was introduced using Open-Babel 3.1.1 software and maintained throughout the simulations. The long-range electrostatic interactions were treated using the particle-mesh Ewald (PME) method [44]. Periodic boundary conditions were applied in all three dimensions.

**Figure 1:**
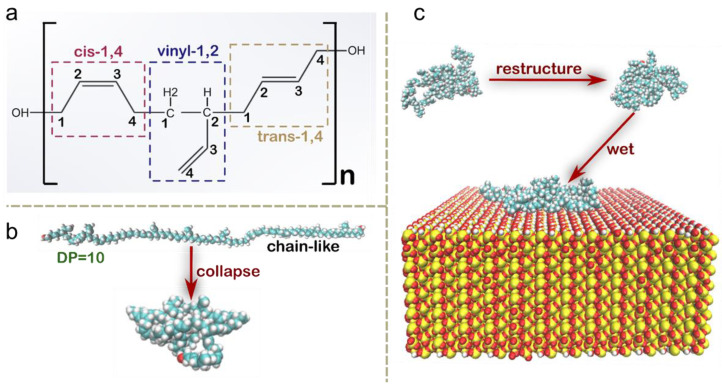
The structure and material surface wetting of HTPB. (**a**,**b**) The chemical structure (**a**) and the molecular structure (**b**) of the HTPB molecule, with a representative depiction of a molecule with a degree of polymerization (DP) of 10. (**c**) Representative snapshots from the simulations showcasing a HTPB molecule with free chains, with restructured chains, or after adsorption onto a quartz surface measuring 10 × 10 nm^2^. Note that the box size in the z direction has been set as sufficiently large to prevent any molecular contact with another surface.

The structure of a single HTPB molecule with varying degrees of polymerization (DP) is depicted in Figure 1a, comprising the cis-1,4/1,2-vinyl/trans-1,4 configuration with a ratio of 1:1:1. In the simulations, the HTPB chain was initially subjected to energy minimization using the steepest descent method to prevent any unreasonable atomic contacts. Subsequently, the system was allowed to relax for 200 ps, leading to a spontaneous transition of the HTPB chain into a low-energy collapsed conformational state, as illustrated in Figure 1b. Following equilibrium attainment, the HTPB chain was positioned at a distance of 5 nm above the surface, and molecular dynamics (MD) simulations were conducted to simulate the spontaneous wetting process of HTPB on the quartz surface (Figure 1c). A time step of 2 fs was employed in these simulations, and molecular trajectories were recorded every 10 ps. For each system under investigation, at least two parallel simulations were performed.

## 3. Results and Discussion

### 3.1. Molecular Conformational Transitions of HTPB During Its Surface Wetting Process

**Figure 2:**
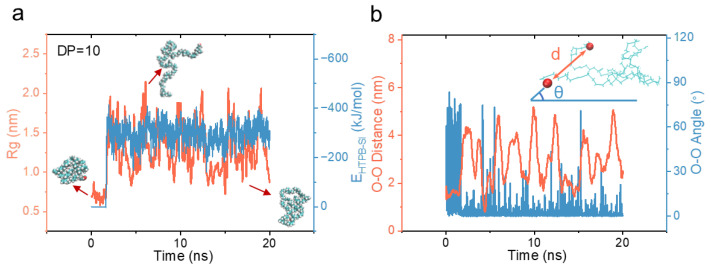
The wetting process of HTPB on the quartz surface. (**a**) The time evolution of the radius of gyration (Rg) of HTPB molecules on the quartz surface, along with their interaction energy. Molecular conformation snapshots at representative time points are shown as insets. (**b**) The corresponding time evolution of the distance between the two oxygen atoms at the polymer’s tip and their angle relative to the substrate surface. The inset illustrates the definition of the O–O distance. DP = 10.

To elucidate the surface wetting behavior of HTPB, our initial focus was on the wetting process of a single HTPB chain with a degree of polymerization (DP) of 10 on a quartz substrate. We examined the time evolution of the interaction energy between the HTPB molecule and the quartz substrate, which revealed a distinct increase between an initial stable stage and an equilibrium plateau, indicating the adhesion behavior of HTPB on the substrate (Figure 2a). Notably, during this interaction process, we observed a noticeable structural transition in the HTPB molecule: prior to contact with the surface, its mean-square radius of gyration (Rg) displayed relatively low values; however, upon adhesion to the surface, the Rg significantly increased. Subsequently, dynamic equilibrium with strong fluctuations occurred. By analyzing molecular conformations at different stages of this process, we discovered that initially there was a collapsed conformation of the polymer chain in the solvent. However, during surface wetting, complex conformational changes occurred in the polymer. A transition from a three-dimensional to a two-dimensional conformation for the polymer chain took place while experiencing an increase in the Rg despite reduced spatial freedom. After adsorption onto the surface, the Rg continued to exhibit vigorous fluctuations as it oscillated between stretched and collapsed conformations (Figure 2a). This behavior sharply contrasted with what was observed in the solvent.

To further characterize the fluctuations in the molecular conformation of the surface-bound HTPB, we analyzed two additional parameters: the O–O distance, which refers to the distance between the two oxygen atoms at each end of the polymer chain, and the O–O angle relative to the substrate (as shown in Figure 2b). It is notable that upon adsorption of HTPB on quartz surfaces, these two parameters exhibited a repetitive pattern of fluctuation characterized by an initial increase followed by a subsequent decrease. Furthermore, we observed an inherent correlation between changes in the O–O distance and the O–O angle; specifically, as the O–O distance decreased, indicating a shift toward a collapsed conformation for the polymer chain, there was a consistent increase in the O–O angle, suggesting detachment from the surface resulting in non-coplanarity of both oxygen atoms. Therefore, these recurring alterations reflect an interplay between HTPB’s adhesion to surfaces and its conformational transformation.

### 3.2. The Contributions of Enthalpy and Entropy in Determining the Surface Wetting of HTPB

**Figure 3:**
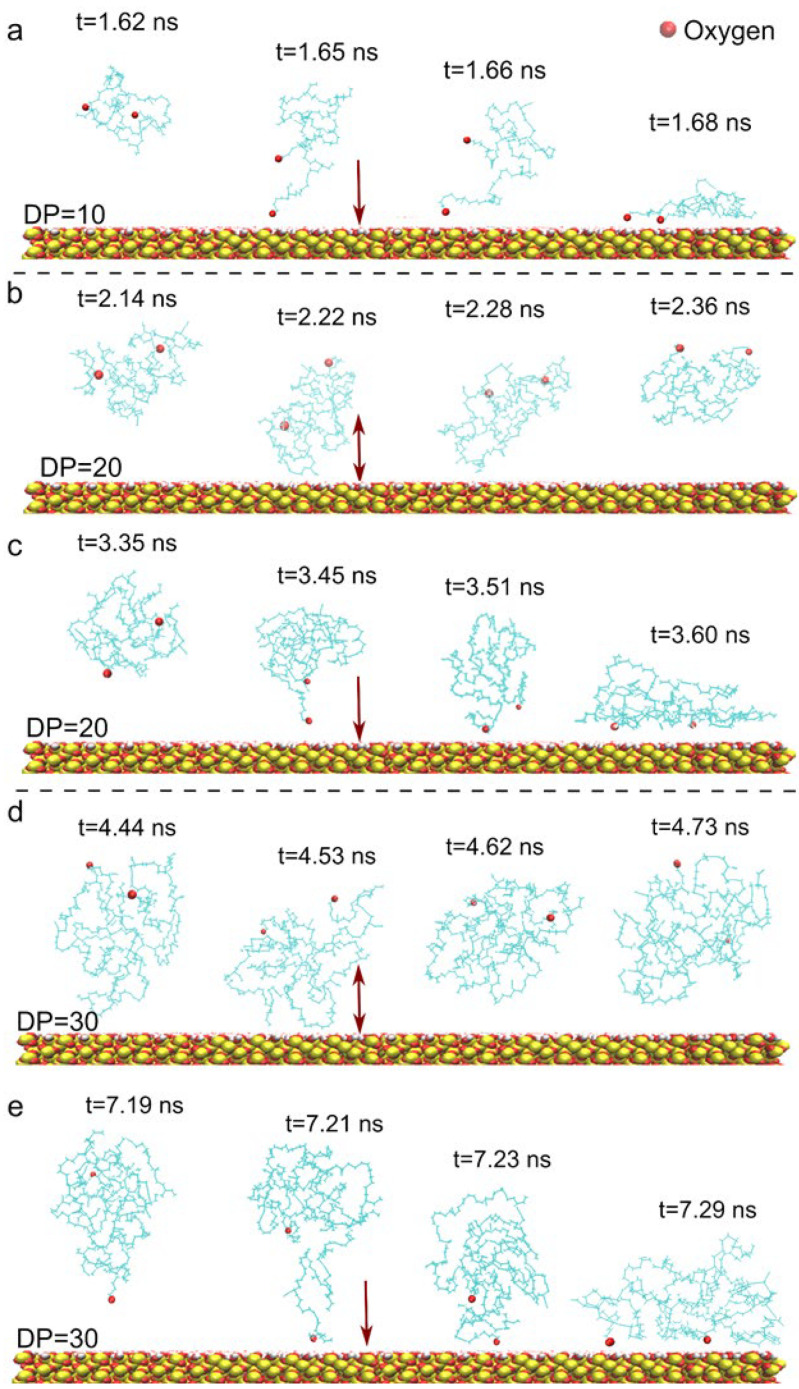
The molecular conformation of HTPB during its interaction with the quartz surface. (**a**,**c**,**e**) The tip-dominant surface adsorption mode of HTPB. (**b**,**d**) An un-adsorption mode of HTPB with an unfavorable conformation. Red arrows denote the subsequent adsorption or aperture of HTPB following instantaneous contact with the surface. For clarification, the oxygen atoms at the end are highlighted with red balls. DP = 10 (**a**), 20 (**b**,**c**), and 30 (**d**,**e**).

By further investigating the interaction process between HTPB and the surface, we have observed several instantaneous contact behaviors of HTPB molecules with the surface. Moreover, it has been determined that the conformation of HTPB at the moment of contact directly influences its subsequent fate, either stable adsorption onto the surface or movement away from it (Figure 3). As depicted in Figure 3b,d, following immediate contact with its side chain, the HTPB molecule tended to move away from the surface and remain in the solvent. It underwent conformational fluctuations despite no significant changes occurring in the Rg value due to its free three-dimensional conformation. In contrast, the HTPB molecule underwent a significant conformational change between stretched and collapsed conformations upon stable adsorption onto the substrate surface, as depicted in Figure 3a,c,e, following initial attachment using its tip. This transformation was accompanied by a distinct alteration in the Rg value, which was quantitatively analyzed and is presented in Figure S1a. A pronounced increase in the energy associated with the interaction between HTPB and the surface, as well as among HTPB molecules, occurred upon contact (Figure S1b). Comparing these two scenarios reveals that differences in molecular conformation upon contact with the substrate primarily account for variations in adsorption effects: the former demonstrated a side-chain-dominant contact mode, whereas the latter exhibited a more intriguing tip-dominant surface adsorption mode. Specifically, initial surface adsorption occurred at one end of the polymer chain where the hydroxyl group is located, facilitating subsequent gradual changes in polymer chain conformation and step-by-step achievement of surface binding. Moreover, when both ends were simultaneously attached to the surface, this process became easier. These phenomena were frequently observed during repeated simulations with different values of the DP (e.g., DP = 20 or 30). These observations unveil an interesting entropy–enthalpy competition mechanism whereby both enthalpy (e.g., polymer–surface adhesion interaction) and entropy (e.g., chain conformational changes) influence HTPB’s wetting behavior on surfaces. This entropy–enthalpy competition mechanism gives rise to the distinct tip-preferential adsorption modes observed in HTPB.

### 3.3. Regulation of the Surface Wetting Effect by Controlling Entropy–Enthalpy Competition

**Figure 4:**
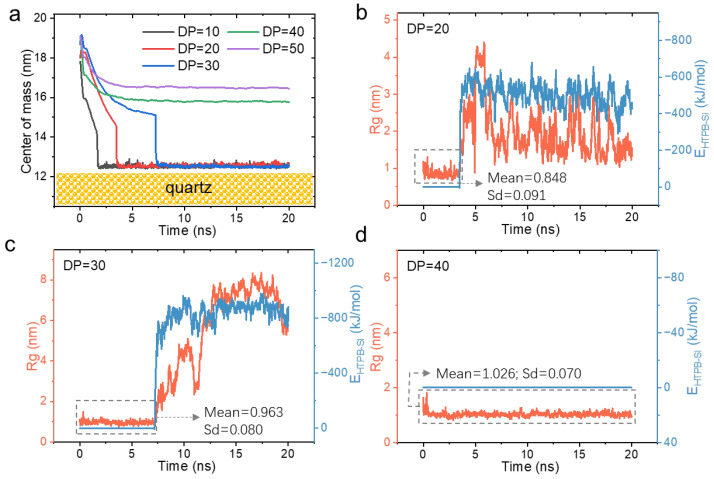
The wetting behavior of HTPB on quartz surfaces with varying degrees of polymerization. (**a**) The comparison of the adsorption process between HTPBs with various DP values. The adsorption process is characterized by the position of the HTPB molecule’s center of mass relative to the quartz surface. (**b**–**d**) The radius of gyration (Rg) and interaction energy between HTPB and quartz during these processes for DP values of 20, 30, and 40, respectively. The mean and standard deviation (Sd) values for the Rg at the stage in solution (marked with dashed gray rectangles) were calculated.

The competition between conformational entropy and adsorption enthalpy offers potential strategies for regulating the surface wetting of HTPB. One approach is to manipulate the conformational entropy of the HTPB molecule, which increases with higher degrees of polymerization [30,45]. Therefore, independent simulations were conducted using HTPB with varying degrees of polymerization, including DP = 10, 20, 30, 40, and 50. Interestingly, Figure 4a reveals a strong length dependence in the surface wetting process of HTPB. Specifically, HTPB with lower degrees of polymerization (e.g., DP ≤ 30) exhibits rapid substrate adsorption within several nanoseconds. Moreover, as the DP increases, the time required for adsorption is significantly prolonged, indicating increased difficulty in surface adsorption for polymers with higher DPs. The previous MD simulations have demonstrated that reducing the chain length is advantageous for the movement of the contact line of n-alkane on α-quartz, thereby confirming an improvement in wettability [37]. Furthermore, experimental measurements of the wetting time validate the finding that aqueous solutions of surfactants achieve optimal wettability with the shortest alkyl chain length in the molecule [46]. Notably, when considering considerably larger DPs (e.g., DP ≥ 40), spontaneous surface adsorption becomes notably more challenging even after extending the simulation time to 40 ns. For molecules with a higher degree of polymerization, both the Rg and the E_HTPB-SI_ (i.e., the interaction energy between HTPB and the quartz surface) increase (Figure S2), indicating that the surface adsorption process requires the overcoming of a greater conformational entropy. These results demonstrate that effective regulation of the surface wetting effect can be achieved by controlling the degree of molecular polymerization.

Furthermore, Figure 4b–d illustrate the conformational changes at the molecular level during the interaction between HTPB and the substrate. Firstly, the molecule demonstrates a more stable Rg value in solution, compared to its adsorbed state on the substrate surface, which exhibits significant dynamic conformational alterations (Figure 4b,c). Secondly, with an increase in the degree of polymerization (e.g., from DP = 20 to 30 and 40), the molecule shows reduced fluctuations in Rg values in solution (dashed gray rectangles in Figure 4b–d), indicating that higher-DP molecules have a greater propensity to collapse into smaller structures. These findings further support our previous conclusion that molecules need to undergo transformation, expose their tips, and bind to the substrate for subsequent stable adsorption.

**Figure 5:**
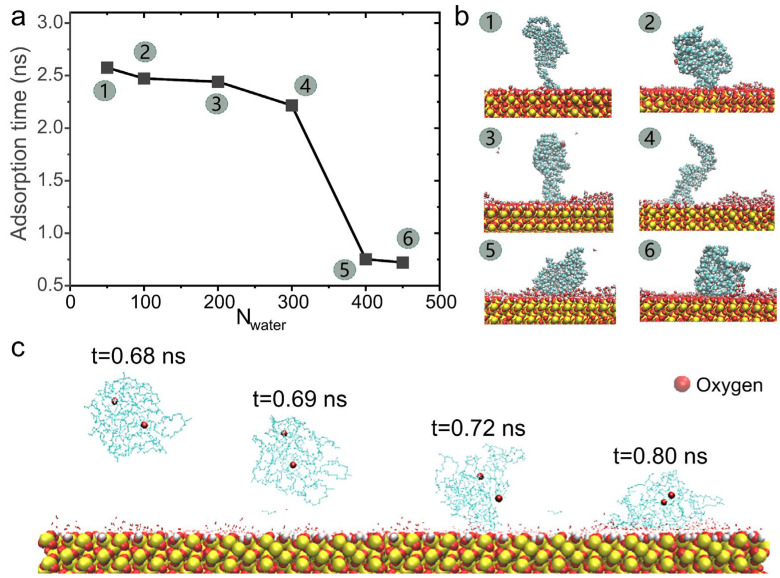
The influence of water number on the surface adsorption behavior of HTPB. (**a**) The temporal distribution of HTPB adsorption onto quartz surfaces with varying water numbers. (**b**) The corresponding representative snapshots. The snapshots were captured at the initial time when the polymer comes into contact with the surface, using a simulation step of 10 ps. (**c**) Representative snapshots demonstrating the conformational changes in the polymer upon contacting the quartz surface, with N_water_ = 400.

Another approach involves regulating the adsorption enthalpy between HTPB and the substrate during their interactions. To demonstrate this, we manipulated the quantity of interfacial water, which refers to water molecules bound to the hydrophilic surface. Water molecules can act as a medium to further enhance the affinity between the substrate surface and the polymer [47]. In this study, we investigated representative wetting processes of HTPB with DP = 50 by varying the amount of interfacial water (N_water_). As depicted in Figure 5a, it was observed that an increase in interfacial water leads to easier surface adsorption of the polymer, as evidenced by a reduction in HTPB’s adsorption time. Therefore, our findings suggest that interfacial water plays a beneficial role in promoting surface wetting of the HTPB polymer.

Intriguingly, our simulations have revealed that the occurrence of surface wetting of HTPB is contingent upon a threshold number of interfacial water molecules (i.e., N_water_ ≈ 350; see Figure 5a). The emergence of this critical value directly correlates with the conformation of HTPB adsorption on the substrate surface. Figure 5b illustrates the molecular conformation of the polymer when it comes into contact with the surface. In cases where there are fewer water molecules present (e.g., N_water_ ≤ 300), HTPB tends to bind to the surface primarily at its tip, followed by gradual adsorption of the molecule. However, when the number of interfacial water molecules surpasses this threshold value (e.g., N_water_ ≥ 400), a noticeable transition in polymer behavior occurs, shifting from a tip-dominant mode for surface wetting to a rapid adsorption mode involving the entire polymer chain. More detailed information can be found in Figure 5c, which demonstrates that under these conditions, the polymer attaches itself entirely onto the water-coated surface without requiring tip adsorption. These observations highlight how interfacial water can serve as an effective means to regulate and modulate subtle entropy–enthalpy competition during HTPB’s process of surface wetting.

## 4. Conclusions

The wetting process of HTPB at the quartz surface was investigated using molecular dynamic simulations in this study. A tip-dominant surface adsorption mode of HTPB was identified, where the hydroxyl group at the end of the polymer chain consistently binds to the surface to initiate the wetting process. Such a finding in our simulations is helpful to understand the unique wetting mechanism of a long polymer chain. Moreover, it was further observed in our simulations that a significant transition in HTPB’s polymer chain conformation occurred during the surface adsorption process, serving as an indicator of successful adhesion between the polymer and the surface. Additionally, during surface adsorption, there is repetitive alteration between stretched and collapsed conformations exhibited by the polymer, resulting in strong fluctuations in its Rg value. These simulation results indicate that both enthalpy (i.e., polymer–surface adhesion energy) and entropy (i.e., polymer conformation entropy) play complex roles in influencing the interaction process or the wetting behavior of polymers on surfaces. It is worth noting that this underlying entropy–enthalpy competition mechanism is crucial for understanding the surface wetting of a polymer chain, which has been somewhat overlooked in previous studies. In addition, based on this enthalpy–entropy competition mechanism, two strategies were employed to modulate HTPB’s surface wetting process: one involved increasing its degree of polymerization to change its conformational entropy, leading to increased difficulty in achieving surface wetting for HTPB; the other strategy focused on regulating the amount of water molecules bound to surfaces and proved effective in adjusting the affinity between polymers and surfaces. Interestingly, it has been demonstrated that there exists a threshold number of interfacial water molecules necessary to achieve proper wetting behavior of HTPB on surfaces (i.e., 350 molecules on a 10 × 10 nm^2^ surface). An increase in the interfacial water content results in a transition from tip-dominant mode to rapid adsorption involving the entire polymer chain. It is important to note that due to computational limitations, the current simulations only investigate small temporal and spatial scales, focusing primarily on the simplest components of the simulation system. However, other intriguing factors such as the impact of HTPB concentration or the influence of additional reaction agents (e.g., curing and coupling agents) leading to cross-linked network formation on the surface also warrant further attention. Overall, our findings provide valuable insights into the surface wetting mechanism of HTPB and propose an entropy–enthalpy strategy for modulating similar behaviors in polymers.

## Data Availability

The data are contained within the article.

## Supplementary Materials

The following supporting information can be downloaded at: https://www.mdpi.com/article/10.3390/polym16213085/s1, Figure S1: The evolution of the radius of gyration components (Rg-X, Y, Z; a) and the interaction energy between HTPB and quartz (E_HTPB-SI_; black) as well as between HTPB and HTPB (E_HTPB-HTPB_; red). DP = 30; Figure S2: Dependence of Rg and EHTPB-SI on DP. Note that for DP ≥ 40, there is no occurrence of spontaneous surface adsorption of HTPB.
