# Supplementary material for: Surface Wetting Behaviors of Hydroxyl-Terminated Polybutadiene: Molecular Mechanism and Modulation
Source: Polymers (Basel). 2024 Oct 31;16(21):3085. doi: 10.3390/polym16213085 (PMC11548704; doi:10.3390/polym16213085)
Supplement: Supplementary file 1 [file polymers-16-03085-s001.zip › polymers-3261329-supplementary.pdf]

polymers

MDPI

Supplementary Material

Surface Wetting Behaviors of Hydroxyl-Terminated Polybutadiene: Molecular Mechanism and Modulation

Xinke Zhang, Zhikun Liu, Bing Yuan * and Kai Yang *

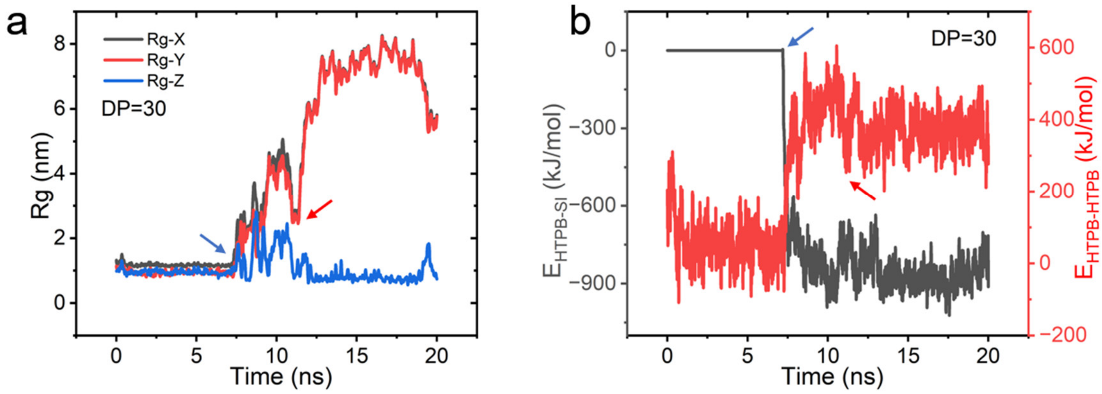

Figure S1. The evolution of the radius of gyration components (Rg-X, Y, Z; a) and the interaction energy between HTPB and quartz ($E_{HTPB-SI}$; black) as well as between HTPB and HTPB ($E_{HTPB-HTPB}$; red). DP = 30.

Corresponding note

To gain a more comprehensive understanding of the role played by enthalpy and entropy in determining the surface wetting behavior of HTPB, we conducted further calculations to track the evolution of various components of the Rg, as well as the interaction energies between HTPB and both the surface and other HTPB molecules (DP = 30). It is evident that significant changes occur in all three components of the Rg at the moment of contact between HTPB and the surface, followed by an observed anisotropic conformational change in HTPB (Figure S1a). Simultaneously, strong interactions are established between HTPB and the surface, as evidenced by a pronounced increase in the energy associated with their interaction ($E_{HTPB-SI}$; Figure S1b). Additionally, there is an increase in its own interaction energy ($E_{HTPB-HTPB}$) upon contact (Figure S1b). Furthermore, it is noteworthy that alterations in the Rg align with corresponding changes in the $E_{HTPB-SI}$ or the $E_{HTPB-HTPB}$ (indicated by arrows in Figure S1a,b). Consequently, these variations observed in the Rg and the interaction energy collectively indicate an interplay between entropy and enthalpy during the process of surface wetting involving HTPB.

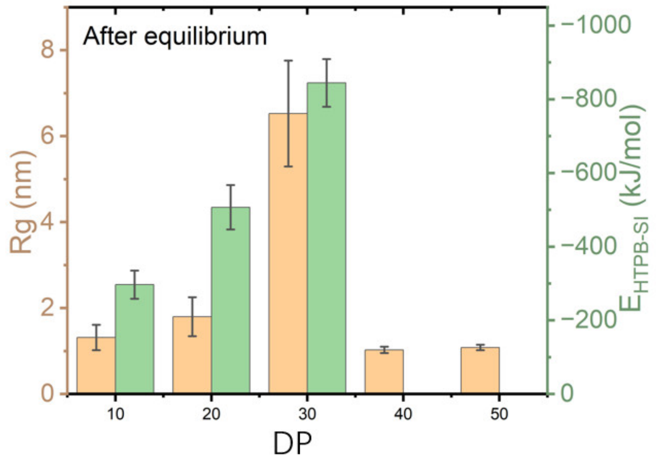

Figure S2. Dependence of Rg and $E_{HTPB-SI}$ on DP. Note that for DP ≥ 40, there is no occurrence of spontaneous surface adsorption of HTPB.

Corresponding note

The competition between entropy and enthalpy can also be observed through the changing trends of the Rg and the $E_{HTPB-SI}$ with the DP (Figure S2): as the DP increases, both the Rg and the $E_{HTPB-SI}$ increase, indicating a significant energy requirement for HTPB to interact with the surface in order to overcome potential conformational changes in longer polymer chains. Furthermore, as the DP continues to increase, the strength of the $E_{HTPB-SI}$ becomes insufficient to compete against the entropy associated with polymer conformation. Consequently, spontaneous surface adsorption of HTPB is hindered.
